# Recent Records on Bacterial Opportunistic Infections via the Dietary Route

**DOI:** 10.3390/microorganisms12010069

**Published:** 2023-12-29

**Authors:** Franca Rossi, Serena Santonicola, Carmela Amadoro, Lucio Marino, Giampaolo Colavita

**Affiliations:** 1 Istituto Zooprofilattico Sperimentale dell’Abruzzo e Molise (IZSAM), Teramo, Diagnostic Laboratory, 86100 Campobasso, Italy; l.marino@izs.it; 2 Dipartimento di Medicina e Scienze della Salute “V. Tiberio”, Università degli Studi del Molise, 86100 Campobasso, Italy; serena.santonicola@unimol.it (S.S.); carmela.amadoro@unimol.it (C.A.); colavita@unimol.it (G.C.)

**Keywords:** dietary route, bacteria, opportunistic pathogens, recent infection records, risk factors

## Abstract

This narrative review was aimed at identifying the opportunistic bacterial pathogens that can be transmitted by contaminated food and represent a current threat for patients particularly susceptible to infections because of underlying conditions or predisposing factors. The analysis was focused on recent case or outbreak reports and systematic reviews published in the years 2019 to 2023 and resulted in sorting 24 bacterial groups comprising the genera or species able to cause a variety of systemic or invasive infections if ingested with food or drinking water. These included both bacteria known to cause mild infections in immunocompetent persons and bacteria considered to be innocuous, which are used in food fermentation or as probiotics. No recent cases of infections transmitted through dietary routes were reported for the critical nosocomial pathogens widely found in food products, primarily *Acinetobacter baumannii* and *Klebsiella pneumoniae*. However, the very first sources of their introduction into the clinical environment still need to be established. In many instances, risky dietary habits, such as eating raw fish, seafood, raw meat, unpasteurized milk, and their derived products or the lack of control in fermentation processes, has led to the reported illnesses, pointing out the necessity to improve the hygiene of production and consumer awareness of the risks.

## 1. Introduction

The definition of opportunistic infection is “a serious, usually progressive infection by a microorganism that has limited or no pathogenic capacity under ordinary circumstances, but which has been able to cause serious disease as a result of the predisposing effect of another disease or of its treatment” [1].

Different bacterial groups present in food or drinking water, other than the major pathogens which are objects of specific surveillance and control measures according to food legislation norms, may behave as opportunistic pathogens in people with underlying conditions or predisposing factors. These belong to bacterial genera or species that cause mild or no illness in immunocompetent persons but can cause life-threatening infections in vulnerable subjects. As an example, lactobacilli, which are essential for food fermentation and probiotics with in vivo proven beneficial effects, were the cause of bacteremia, endocarditis, and other localized infections most often in immunocompromised, diabetic persons, or patients with a history of predisposing events such as medical interventions, diseases, or oral infections and dental procedures [2,3]. 

The aim of this analysis of the scientific literature was to offer an overview of the risks posed by dietary sources in the transmission of opportunistic bacterial pathogens and obtain indications for their prevention. The reports indicating a possible or ascertained origin of an opportunistic bacterial infectious agent from food or drinking water were included with no restrictive criteria.

A preliminary search of the literature sources was carried out in Google Scholar (https://scholar.google.com/schhp?hl=it, accessed on 15 November 2023) using the keywords “opportunistic AND pathogen AND human AND food AND infection” limited to the last five years. This search retrieved 17,500 results that were ordered by pertinence. The first five hundred items were checked for the names of pathogens to be specifically evaluated for their involvement in infections of dietary origin, resulting in the identification of 42 bacterial genera or species. These were reduced to 24 after a second search in which the retrieved bacterial names were individually used in the keyword associations “organism name AND food AND human AND infection AND case” or “organism name AND food AND human AND infection AND outbreak”. This second search allowed us to retrieve reports that indicated the oral route as the most probable source of infection after screening the content of the first one hundred items obtained for each organism name ordered by pertinence. The same search strategy was also used in the PubMed (https://pubmed.ncbi.nlm.nih.gov/, accessed on 18 November 2023) and Scopus (https://www-scopus-com.bibliosan.idm.oclc.org/search/form.uri?display=basic#basic, accessed on 20 November 2023) databases, but did not result in the retrieval of additional items of interest for this survey.

## 2. Recent Cases of Opportunistic Infections Transmitted by Food or Drinking Water

A total of 54 recent cases or outbreak reports were retained for description since they fulfilled the criterion of “infection most likely acquired through the dietary route”. Some cases caused by bacteria whose food origin was well established were included even if a probable food source was not mentioned in the report. 

The reports described in this review are summarized in Table S1 [4,5,6,7,8,9,10,11,12,13,14,15,16,17,18,19,20,21,22,23,24,25,26,27,28,29,30,31,32,33,34,35,36,37,38,39,40,41,42,43,44,45,46,47,48,49,50,51,52,53], where the bacterial groups involved in the illnesses are reported in alphabetical order along where the type of illness, source of infection, underlying diseases or predisposing condition/s, bibliographic sources, method of identification of the etiological agent, and antibiotic resistance (AR) profile are shown. The order of the listing within the bacterial genus is chronological.

The food sources involved in opportunistic infection transmission and the bacterial taxa implied are listed in Table 1.

The recent reports retrieved for each group of opportunistic bacterial pathogens possibly acquired through the dietary route and the literature on their relevance to public health are summarized in the following sections.

### 2.1. Aeromonas spp.

The genus *Aeromonas* comprises Gram-negative gas-producing bacilli belonging to the class Gammaproteobacteria, classified in 36 species. Among these, *Aeromonas hydrophila*, *A. caviae*, *A. dhakensis*, *A. veronii*, *A. salmonicida*, and *A. sobria* are known to cause human infections mainly in the form of mild gastroenteritis [4,54]. These bacteria can be isolated from fresh water and sea water, soil, fish, meat, and other foods. The major virulence factors of *Aeromonas* spp. are hemolysins, enterotoxins, invasins, aerolysin, adhesins, proteases, phospholipase, and lipase [4]. 

*A. sobria* may act as an opportunistic pathogen and cause bacteremia, intestinal, and extraintestinal infections predominantly in patients with chronic hepatic disease, gastroenteritis, malignancy, and an immunocompromised status. Gastroenteritis is the most common infection caused by *Aeromonas* spp., but peritonitis is not uncommon, especially in patients with cirrhosis [4]. 

*A. sobria* caused peritonitis in a 37-year-old man affected by renal failure and under peritoneal dialysis (PD). He was admitted to the hospital with a fever, vomiting, abdominal pain, diarrhea, and cloudy dialysate several hours after eating stinky tofu. Stinky tofu, a kind of traditional Chinese food, is usually considered unhygienic for the particular production process in which the tofu is placed in water for a long time to increase the unique smell. Therefore, it was speculated that the stinky tofu was the source of infection in this case. Bacterial translocation through the intestinal barrier plays an important role in the pathogenesis of PD-related peritonitis. Amikacin and levofloxacin treatment allowed for the patient to recover [4].

*A. hydrophila* causes acute gastroenteritis or diarrhea most often through the ingestion of infected fish and seafood with an incubation period of less than 24 h. A case of gastroenteritis complication involved a 74-year-old woman admitted to the hospital with worsening epigastric pain, vomiting, and diarrhea that started 3 days previously after eating raw ayu fish. Her husband also had eaten the fish and was affected by mild diarrhea. The woman, who did not present underlying conditions and was not a smoker or alcohol consumer, presented mild renal failure and sepsis. Abdominal computed tomography (CT) revealed branched portal vein gas in the right hepatic lobes and a thickened gastric wall and intestinal edema. A submucosal tumor-like elevation in the posterior wall of the gastric corpus, which contained an ulcer at the center of the lesion, was revealed through digestive endoscopy. *A. hydrophila* was isolated from the stool and identified using microbial identification techniques so the diagnosis was of phlegmonous gastritis caused by *A. hydrophila* with a hepatic venous pressure gradient (HPVG) and without necrosis of the intestinal tract. After treatment with levofloxacin, then switched to cefmetazole, the symptoms gradually improved. In this case, the predisposing factor for the *A. hydrophila* infection presentation was most probably the presence of an ulcer lesion in the stomach that favored the invasion of the gastric wall and phlegmon formation [5].

### 2.2. Acinetobacter spp. 

The genus *Acinetobacter* currently comprises approx. 80 species of strictly aerobic Gram-negative coccobacilli [55]. *A. baumannii* is the species most often involved in infections, but also cases attributed to *A. calcoaceticus*, *A. lwoffii*, *A. haemolyticus*, *A. johnsonii*, *A. junii*, *A. nosocomialis*, *A. pittii*, *A. bereziniae*, *A. serfertii*, *A. schindleri*, and *A. ursingii* were described. The community-acquired infections reported for *A. baumannii* include respiratory infections in children, immunocompromised individuals, and patients with risk factors such as alcoholism, smoking, and diabetes mellitus as underlying conditions [56]. 

The presence of this genus, mainly represented by the species *A. baumannii*, *A. calcoaceticus*, and *A. lowfii*, in milk, even pasteurized, dairy products, bacon, eggs, chicken, fish, fresh meat, fresh fruits, and produce is well established based on reports from different countries [55,57,58]. In the latter food category, these bacteria may persist after mild disinfection with vinegar or hypochlorite [58]. Moreover, *A. lwoffii* and *A. johnsonii* can survive in a wide range of temperatures, low pH values, and are resistant to disinfectants, irradiation, and desiccation [57].

However, the relevance of *A. baumannii* as an opportunistic pathogen is mainly attributable to nosocomial infections that can be transmitted from one patient to another as a consequence of environmental contamination transferred to medical devices, such as tubes and catheters, as observed in COVID-19 patients [56,59]. 

*A. baumannii* carbapenem resistant variants (CRAB) emerge in contexts with a high antibiotic pressure and an underregulated usage of antibiotics [60], which represent the most relevant concern posed by this bacterial species. This is among the six “ESKAPE” pathogens (*Enterococcus faecium*, *Staphylococcus aureus*, *Klebsiella pneumoniae*, *Acinetobacter baumannii*, *Pseudomonas aeruginosa*, and *Enterobacter* species), whose multi-drug resistance and ability to escape antimicrobial treatments make them responsible for the majority of nosocomial infections with a high mortality rate [61]. However, in clinical settings, the origin of the bacterium has remained unknown in many cases [56].

Hospital-acquired *Acinetobacter* infections may occur in patients with malignancies, patients in intensive care units (ICU) and burn units, and patients who have undergone major surgical procedures, are neutropenic, or have underlying chronic diseases such as diabetes mellitus and chronic pulmonary diseases [62]. These infections include bacteremia, meningitis, pneumonia, and other infections such as central nervous system infections after neurosurgery. Colistin sulfate, a derivative of polymyxin E, is the last resort drug for treating CRAB infections [63]. The viable but not culturable (VBNC) state was proposed as a persistence mechanism allowing *A. baumannii* for coping with environmental stresses [64].

An outbreak of *A. baumannii* infections that occurred through ingestion was reported in mechanically ventilated patients in an emergency ICU who received oral care after drinking water from contaminated taps [65].

Recent reports on the direct involvement of the dietary route in the transmission of *Acinetobacter* spp. are lacking and one retrieved report was published in 2009 [57]. The case regarded the gastrointestinal symptoms and bacteremia caused by *A. lwoffii* in a previously healthy 64-year-old man who had dined in two different restaurants the day before hospitalization. The absence of a catheter line, which is usual in *Acinetobacter* spp. infections, led the researchers to suspect food as source of the infectious agent. 

### 2.3. Arcobacter spp.

*Arcobacter* spp. is a genus of aerotolerant, Gram-negative bacteria of the class Epislonproteobacteria and the family Campylobacteraceae that can grow at temperatures higher than 30 °C and comprises more than 30 known species. Four of these have been reported to infect humans, namely *Arcobacter butzleri*, *A. cryaerophilus*, *A. skirrowii*, and *A. thereius* [6,66]. 

*A. butzleri* was first isolated in aborted bovine fetuses and was later linked to reproductive disorders and late-term abortions in cattle, pigs, and sheep. It can be present in a range of commonly consumed meat products [7] and bovine milk [67].

*Arcobacter* spp. was indicated by the European Food Safety Authority (EFSA) as “an issue that deserves further attention in terms of the burden of disease as it is most probably underreported”. It was mentioned for the first time in 2021 by the International Health Intelligence (IHI) platform for a prevalence study in bivalve mollusks carried out in Sardinia, Italy [68]. An infection cluster involved eight patients treated for acute diarrhea in a tertiary hospital in Cantabria, Northern Spain. The *Arcobacter* infection originated from different sources since the patients belonged to three categories, namely elderly persons, persons recently returned from journeys abroad, and persons with comorbidities. Fingerprinting methods indicated no clonal relationships among the isolates, which were all identified as *A. butzleri.* In this study, the possible dietary sources for *A. butzleri* were not investigated but the AR profiles were obtained, which indicated a resistance to cefazolin for all eight isolates. A resistance to amoxicillin–clavulanic acid and tetracycline was observed for five and four isolates, respectively [66].

Several sporadic outbreaks of *A. butzleri* gastroenteritis were observed in the U.S., Europe, and South Africa in the years 1990 to 2000. *A. butzleri* was identified as a causative organism for 24 out of 4636 cases of gastroenteritis in a prospective study in Germany, while several studies have identified *A. butzleri* to be among the most frequently isolated Campylobacteraceae in human clinical samples [6,7]. Persistent watery diarrhea is the main symptom of *Arcobacter* spp. infection, though bacteremia and septicemia were also reported. The prevalence of this emerging pathogen is not well documented due to the difficulty of its identification in clinical settings [6]. 

According to a phylogenetic analysis of the isolates from Thailand, the *A. butzleri* STs were defined by sequencing seven housekeeping genes, *asp*A, *atp*A, *gln*A, *glt*A, *gly*A, *pgm*, and *tkt.* Those that are more often involved in human infections are ST94 and ST166, which have been found in both human diarrheal stool samples and chicken offal or meat samples [69].

A case of pericarditis caused by *Arcobacter* spp. was reported for a 32-year-old male with a past medical history of well-controlled human immunodeficiency virus (HIV), antiviral therapy, and end-stage renal disease (ESRD) admitted to internal medicine for COVID-19 pneumonia. This patient presented worsening cardiac tamponade that was successfully managed with urgent pericardiocentesis, steroids, antibiotics, and pericardial drain. The patient reported that, about a month earlier, he had several episodes of diarrhea after consuming chicken from a local restaurant. The cultures of pericardial fluid and blood on aerobic blood agar were positive for Gram-negative rods identified by VITEK 2 as *Arcobacter* species. This case highlighted that COVID-19 infection can increase the risk and severity of secondary bacterial infections by damaging the respiratory tract and compromising the immune system [6]. 

A 38-year-old man with a history of HIV infection arrived at the hospital with symptoms of acute watery diarrhea lasting for two weeks. There was no recent travel history or intake of raw or uncooked food. The stool cultures were positive for *A. butzleri*. The infection was successfully treated with ciprofloxacin but, unfortunately, the patient passed away due to hospital-acquired severe pneumonia. The authors concluded that clinicians should recognize the pathogenicity of *A. butzleri* in immunocompromised hosts [7]. 

### 2.4. Bacillus spp.

*Bacillus* strains belonging to the species *B. subtilis*, *B. amyloliquefaciens*, *B. licheniformis*, *B. circulans*, *B. pumilus*, and *B. brevis* are used in Asian and African countries to produce popular fermented foods from different bean types. With the exception of *B. subtilis* natto, a starter used in industrial natto production, all the other *Bacillus* spp. used in these foods are naturally occurring [70]. Moreover, different *Bacillus* species are used as probiotics, including *B. clausii*, *B. coagulans*, *B. licheniformis*, *B. polyfermenticus*, *B. pumilus*, *B. subtilis*, and non-toxigenic strains of *B. cereus* [71].

Two cases of bloodstream infections occurred after the administration of a *B. licheniformis* probiotic preparation to treat gastrointestinal bleeding due to *Clostridium difficile* colitis. One occurred in a 67-year-old woman with COVID-19 infection and hepatic diseases who developed antibiotic-associated diarrhea with positive occult blood. This condition worsened after the administration of capsules with 250 million live *B. licheniformis* twice a day. Moreover, sepsis caused by this organism was observed and, despite the discontinuation of the probiotic therapy, the patient died from severe pneumonia and septic shock due to the ineffectiveness of the therapy with levofloxacin and vancomycin. The other case regarded a 76-year-old woman admitted to a resuscitation unit for respiratory failure caused by pneumonia. This patient had a history of hypertension, coronary artery disease, heart failure, and obesity. She was treated with different antibiotics in succession that caused diarrhea with occult blood, so she was started with the *B. licheniformis* probiotic. While the diarrhea improved, she developed *B. licheniformis* sepsis that was successfully treated with moxifloxacin and vancomycin [8].

In these two cases, whole genome sequencing (WGS) confirmed that the blood isolates were identical to the administered probiotic. Treatment with the *B. licheniformis* probiotic should, therefore, be avoided in patients with intestinal bleeding that indicates a disruption of the mucosal barrier and can determine the translocation of the probiotic in the bloodstream [8]. 

A case of *B. subtilis* variant natto bacteremia was identified in 2021 in a 56-year-old Japanese woman who presented abdominal pain after assuming barium for gastric radiographic examination and used to eat fermented soybeans (natto) daily. A perforation of the sigmoid colon and generalized peritonitis were diagnosed. Sepsis caused by *B. subtilis* was ascertained from the blood cultures and was successfully treated with antibiotics. The *bio*F gene sequence of the blood isolate was 100% identical to the *B. subtilis* var. natto strain. Other cases of *B. subtilis* bacteremia consequent to intestinal perforation were reported previously in Japan and this constitutes to be a risk related to the consumption of fermented products containing this bacterial strain [9]. 

A case of infection caused by *B. pumilus* involved a 51-year-old Japanese man who started to experience strong chills some hours after eating reheated rice and stewed minced meat from a Kenyan restaurant. He recovered after 2 days of therapy with probiotics and ciprofloxacin. The blood cultures were positive for *B. pumilus* identified using physiological standardized tests and 16S rDNA sequence analysis. Previous studies reported that meat dishes, eggs, baked products, and canned tomato juice were involved in presumptive food poisoning by *B. pumilus* and that this bacterium can produce a heat-stable toxin, as observed in milk isolates in Finland. Moreover, pre-cooked rice contaminated with the pumilacidin-producing *B. pumilus* strain was implicated in a case of food poisoning in Norway [10].

### 2.5. Burkolderia spp.

The *Burkolderia* genus comprises Gram-negative, rod-shaped, motile bacteria, among which the strains able to cause infections with food as a possible source are mainly attributed to *B. gladioli* and to the *B. cepacia* complex [72,73]. However, no case reports directly linked to food consumption were available for this genus.

### 2.6. Chryseobacterium spp.

*Chryseobacterium indologenes* is a Gram-negative bacterium found in soil, water, plants, and food products for which water systems are a reservoir, and it is not eliminated by chlorination. Therefore, when present in municipal water supplies, it can cause infections such as pneumonia, bacteremia, cellulitis, urinary tract infections, ocular infections, meningitis, peritonitis and, in clinical settings, surgical wound infections and catheter-related infections, also in immunocompetent individuals [74]. The comparison of the antibiotic sensitivity spectra indicated that this bacterium is becoming increasingly resistant to antibiotics. A Taiwanese study reported that 98.8% of *C. indologenes* infections were nosocomial, with a mortality rate of 25%, and 79.8% of the patients had comorbidities [75]. 

### 2.7. Coagulase-Negative Staphylococci

Coagulase-negative staphylococci (CNS) are frequently found in raw fermented meats and cheeses since they are natural colonizers of skin and mucosae in animals and humans [76,77,78]. Among these, *Staphylococcus epidermidis* and, in a minority of cases, *S. capitis*, *S. haemolyticus*, *S. hominis*, and *S. warneri* were responsible for 90% of the cases of late onset sepsis (LOS) in neonatal intensive care units (NICUs) [79]. LOS is defined as a bloodstream infection occurring between 3 and 28 days of life and is caused by bacteria transmitted horizontally in the hospital or community environment [78]. CNS are the most common organisms in mucocutaneous sites and the nasopharynx in the first week of life and the infection risk factors are extreme prematurity and parenteral nutrition with intravascular catheters. The increasing prevalence of CNS infections is attributable to their increasing antibiotic resistance and their ability to produce biofilms [79]. 

CNS has been recognized as a cause of infective endocarditis in humans since the 1980s. While CNS endocarditis mostly occurred in patients with prosthetic valves and in situ grafts, there was a notable increase in the incidence of CNS-induced native valve endocarditis. Rare cases of right-sided infective endocarditis (RSIE), which affects the tricuspid or pulmonary valve, have been reported after septic abortion, abscess, and septic arthritis [80].

*S. saprophyticus* is the cause of uncomplicated urinary tract infections (UTIs) in 10–20% of young women with a high recurrent infection frequency. Rare complications include acute pyelonephritis, nephrolithiasis, and endocarditis. Meat and other foods are suspected to be the sources of human gut colonization by *S. saprophyticus*. From the genomic data of 321 human UTI isolates collected from eight countries in four continents during 1997–2017, two lineages, G and S, with distinctive evolutionary histories, were identified, enriched in different sets of genes, and both disseminated geographically. The high relatedness of the UTI isolates from different patients in the same country and in different countries led to the hypothesis that these could belong to a cross-border chain of transmission through a food source [81].

Pork is frequently contaminated with *S. saprophyticus*, which was found in slaughterhouse samples, including the meat, equipment, and workers’ hands, and most likely introduced by live pigs. The comparison of 104 isolates collected from a slaughterhouse and 128 isolates collected from human UTIs in Lisbon during 2016–2017 showed that all the isolates belonged to a single lineage (lineage G). The phylogenetic reconstruction of all the isolates based on a single nucleotide polymorphisms (SNPs) analysis showed examples of the relatedness of the slaughterhouse and human isolates. 

The genes that seemed to be associated with an increased pathogenic potential of *S. saprophyticus* were those encoding a SplE-like protein and a gene cluster encoding a complete accessory secretory system associated with a serine-rich adhesion similar to SraP. In addition, the genes encoding resistance to the antimicrobial drugs trimethoprim (*dfr*G), lincosamide (*lnu*A), streptogramin B (*erm*44v), or macrolides (*mph*C-*msr*A) were also associated with the strains from human infections [81].

### 2.8. Comamonas spp.

*Comamonas testosteroni* is an aerobic Gram-negative bacillus, previously denominated *Pseudomonas testosteroni*, that is able to use testosterone as a sole carbon source. It is commonly found in soil, plants, wastewater/sludge, fresh water, and the animal intestinal microbiome. It was also isolated from activated sludge, polluted soil, hospital environments, and clinical samples [11,82]. Rare cases of infection caused by this bacterium include peritonitis, cellulitis, endocarditis, meningitis, pneumonia, endophthalmitis, and tenosynovitis, and were community acquired in most cases. *C. testosteroni* bacteremia recently regarded a 46-year-old Indian woman from a rural area who, differently from the previously reported cases, was immunocompetent and with no underlying conditions. The source of infection was supposed to be contaminated food or water, though this was not proven [11]. 

A recent analysis of the medical reports highlighted that other species, such as *C. kerstersii*, *C. aquatica*, *C. thiooxydans*, and *C. terrigena*, can also cause infections that are on the rise together with increased antibiotic resistance [82]. 

### 2.9. Enterobacter spp.

The *Enterobacter cloacae* complex (ECC) comprises different species of Enterobacteriaceae, including *E. cloacae*, *E. mori*, *E. ludwigii*, *E. kobei*, *E. asburiae*, *E. cancerogenus*, and *E hormaechei* [83,84], and is considered to be of low virulence and to cause infections in immunocompromised patients. However, *E. bugandensis* was identified as a hypervirulent species responsible for fatal septic shock in newborns in France. Moreover, an epidemic hypervirulent clone of *E. hormaechei*, sequence type ST133, in 2022 showed a high invasiveness and mortality in clinical settings [82]. This clone was first isolated in the U.K. from blood samples and showed multi-drug resistance (MDR), though it did not carry carbapenemase genes. 

*Enterobacter cloacae* is responsible for human infections, such as pneumonia, urinary tract, skin, soft tissue, and intravascular infections, which are more frequent in patients who underwent antibiotic treatments in ICUs. However, the most severe infection caused by this bacterium is endocarditis that most commonly affects the mitral valve and showed a mortality rate of 30%, according to a recent systematic review. Fever occurred in 92.3% of patients, sepsis in 38.5% of patients, and shock in 25% of patients. The predisposing factors were the presence of a prosthetic valve, intravenous drug use, end-stage renal disease, cardiac surgery within one month, and the presence of a central venous catheter (CVC) [85]. 

A recent Investigation carried out in Spain reported that the presence of bacteria of the *E. cloacae* complex was able to produce AmpC β-lactamases in 10.2% of the samples of fresh vegetables intended for raw consumption. In two isolates from tomato and irrigation water, extended-spectrum β-lactamases (ESBL) and carbapenemase were also found. AmpC-positive isolates, without the coproduction of other important β-lactamase enzymes, are controlled by cefepime and carbapenems. However, the isolates of *E. hormaechei*, an emerging human pathogen, were not inhibited by cefepime. Such strains might represent a direct public health concern through the consumption of fresh vegetables [84]. 

### 2.10. Enterococcus spp.

The genus *Enterococcus* comprises lactic acid bacteria (LAB) that commonly inhabit the guts of humans and animals. *Enterococcus faecium* and *E. faecalis* are the species most often encountered in various fermented foods. Some *Enterococcus* strains are utilized as probiotics and some produce bacteriocins with promising application perspectives for food safety improvement. However, enterococcal strains can carry and easily transfer plasmid-mediated antimicrobial resistance genes that have contributed to the emergence of vancomycin-resistant enterococci (VRE), listed by the WHO in the Priority 2 level of bugs requiring special surveillance [86]. Moreover, some *E. faecalis* strains can carry genes for the production of a two component cytolysin that has been proven to increase the severity of liver disease and mortality in patients with alcoholic hepatitis [87].

There were two recent reports on the ascertained or presumptive involvement of these bacteria in foodborne infections. One regarded a 59-year-old hemodialysis patient with a 5-day history of fever and diarrhea, which were possibly caused by the ingestion of contaminated food. *E. casseliflavus* was isolated from the blood cultures and upper abdominal magnetic resonance imaging (MRI) revealed a polycystic liver and kidney, and some cysts with potential bleeding as a complication possibly caused by the infection. Additionally, the cyst fluid culture was positive for *E. casseliflavus*, confirming that the patient had both bacteremia and a liver cyst infection caused by this bacterium. Long-term dialysis is a known risk factor for colonization by *E. casseliflavus*, and ampicillin associated with gentamicin or streptomycin is considered a standard treatment. The patient was treated with meropenem and β-lactamase inhibitors, but the infection remained difficult to control possibly because it was internal to the hepatic cyst. A better outcome was indeed achieved after the cyst was drained [12]. 

Another report regarded an outbreak of gastroenteritis presumably caused by drinking water contaminated by *E. faecalis*. The clinical signs were abdominal pain, vomiting, fever, and diarrhea, which varied among 690 patients who required treatment in the emergency room at the Kruja Hospital, Albania. The analysis of the water samples analyzed by the National Institute of Public Health were found to be contaminated with *E. faecalis*. The enterococcal isolate from the water was not characterized further. Only 22 stool samples of the patients were analyzed, of which two were positive for Norovirus G2 and one among ten tested was positive for *Salmonella* species. The occurrence of these two pathogens was considered coincidental and not linked to the outbreak. The interruption of the drinking water supply led to the cessation of clinical cases [13]. 

### 2.11. Klebsiella spp.

The genus *Klebsiella* belongs to the Enterobacteriaceae family and was included by the WHO among the most critical group of multi-drug-resistant bacteria that pose a particular threat in hospitals, nursing homes, and among patients whose care requires devices such as ventilators and blood catheters [88,89]. Antibiotic resistance is frequent within the genus and the plasmid-mediated spread of genes encoding carbapenemases is of particular concern since the clones of *K. pneumoniae* and other *Klebsiella* species carrying these genes are very frequent in non-clinical contexts, including livestock and wastewater. A One Health approach to investigate the public health risks posed by the non-clinical reservoirs of antibiotic resistance is, therefore, needed.

A large-scale study was based on the WGS data for 3482 isolates recovered from 6548 clinical, community, veterinary, agricultural, and environmental samples collected around the city of Pavia, in Northern Italy, within a 17-month period. The isolates were assigned to 15 *Klebsiella* species, including *Raoultella*, a genus most probably invalidly separated from *Klebsiella*. Approximately half of the isolates were identified as *K. pneumoniae*. This unprecedented sampling and sequencing effort within a restricted geographical area that is a known hotspot for healthcare-associated multi-drug-resistant *K. pneumoniae* led to the observation of low levels of resistance and virulence genes outside the clinical settings and in species other than *K. pneumoniae*, suggesting that the emergence of highly virulent and/or resistant lineages within the environment is rare. Moreover, the data showed the emergence of potentially high-risk lineages within the hospital setting for *K. pneumoniae* and other species, such as the newly described lineage *K. quasipneumoniae* ST571. Since the analysis revealed that transmission is much more common within than between settings, it was concluded that the *Klebsiella* spp. niche adaptation plays a role in mitigating transmission from animal and environmental sources to humans [88]. 

However, *K. pneumoniae* infections of food origin, though occurring in a clinical setting, were reported. An outbreak was caused by a *K. pneumoniae* clone producing CTX-M-15 extended-spectrum β-lactamase in a NICU in Norway, where 58 children were colonized but only one developed bacteremia. The *K. pneumoniae* strain was probably introduced from the breast milk of one mother [14].

Community-acquired *K. aerogenes*, formerly *Enterobacter aerogenes*, infections are rare, while most reported cases were associated with hospital-acquired infections. A case of a urinary tract infection (UTI) occurred in a 63-year-old woman from Bangladesh who was probably infected from contaminated water while working in the house. The patient had an history of type 2 diabetes and hypertension. The infection with water as the source was considered possible since drinking water from tube wells, which is common in Bangladesh, was previously shown to be contaminated by *K. aerogenes*. The genome sequence showed that the isolate was multi-drug resistant for the presence of 17 antibiotic resistance genes to aminoglycosides (*aph*(3′)-Ib, *aph*(6)-Id, *aac*(3)-Ile, *aac*(6′)-Ib-cr), β-lactams (*bla*TEM − 1B, *bla*CTX − M−15, *bla*OXA − 1, *blaamp*C), fluoroquinolones (*oqx*A, *oqx*B), amphenicol (*cat*B3), fosfomycin (*fos*A), and tetracyclines (*tet*D). Folate pathway antagonists, the resistance genes *dfr*A14 and *sul*2, and the efflux pumps *acr*AB associated with tigecycline resistance were also present. Indeed, the strain was resistant to aminoglycosides, penicillin, cephalosporins, amphenicol, fluoroquinolones, folate drugs, tetracyclines, phosphonic acid, and glycycline (Table S1). However, it was susceptible to carbapenems and polymyxins. After the 14th day of antibiotic administration, the patient recovered completely [15]. 

### 2.12. Lactobacilli

The role of lactobacilli as opportunistic pathogens was summarized in recent reviews that illustrated the routes of infection, types of illnesses caused, and some genetic traits that could discriminate potentially pathogenic strains [2,90]. However, in the past year, another seventeen case reports of infections caused by lactobacilli, of which nine had a link to probiotic or yogurt ingestion, were published. It must be underlined that the new nomenclature of lactobacilli approved since 2020 is still not in use in clinical reports, indicating difficulties in the application of taxonomic updates in clinical practice. 

A 76-year-old woman with a medical history of atrial fibrillation, congestive heart failure, type 2 diabetes, and hypertension was hospitalized for acute and chronic systolic heart failure and cholelithiasis with possible cholecystitis and gallbladder wall thickening. The fluid obtained through the percutaneous drainage of the gallbladder was positive for *Lacticaseibacillus paracasei*. The patient underwent antibiotic treatment, but cholecystectomy was not carried out because of her critical condition. The increased intake of yogurt by the patient may have possibly caused the infection [16]. 

A man in his late 60s, with a history of moderately severe ulcerative colitis treated with a blend of *L. paracasei*, *L. acidophilus*, *Lactiplantibacillus plantarum*, *L. casei*, *Ligilactobacillus salivarius*, and *L. rhamnosus* had a bioprosthetic aortic valve replacement complicated by acute respiratory distress syndrome requiring tracheostomy and extracorporeal membrane oxygenation. Some months later, he developed a septic shock with a presumed respiratory source of infection. The patient was immunosuppressed for a recent therapy with prednisone. The blood cultures were positive for *L. rhamnosus*, identified using matrix-assisted laser desorption ionization time of flight mass spectrometry (MALDI TOF MS). Bacteremia persisted for three days while the antibiotic therapy was carried out with penicillin and meropenem, later changed to intravenous ampicillin. Post-hospitalization, the patient developed subacute bioprosthetic aortic valve endocarditis and secondary septic emboli to the brain; intravenous gentamycin, and ampicillin allowed for complete symptom resolution. The intestinal translocation of *L. rhamnosus* was possibly favored by the immunosuppression and microbiome-disrupting antibiotic therapy [17].

A 74-year-old man was hospitalized for a fever and severe back pain. He had type 2 diabetes and ischemic heart disease as underlying conditions. He often took laxatives and probiotics for chronic constipation. A histological examination of the lesions involving the L1/L2 vertebrae revealed inflammation and showed the presence of *L. paracasei*, which was also detected in the blood cultures. Treatment with ampicillin and clindamycin healed the infection. Unfortunately, the patient experienced two heart attacks, the first during hospitalization, and the second, which was fatal, after he was discharged [18].

A 22-year-old woman presented native mitral valve endocarditis with severe regurgitation and required a valve replacement. *L. jensenii*, identified using MALDI TOF MS, was isolated from the blood cultures and from the excised valve. She was reported to have regularly consumed probiotic yogurt for chronic constipation in the past years. Antimicrobial therapy with vancomycin and meropenem was effective in eliminating the infectious agent [19].

A 61-year-old immunocompetent woman with uncontrolled diabetes mellitus, non-ischemic cardiomyopathy, ventricular tachycardia, and ventricular fibrillation status post biventricular automated intracardiac defibrillator (AICD) presented nausea, emesis, a systolic murmur, and extensive redness around the upper thigh. She was recently hospitalized for bacteremia and the blood cultures were positive for *L. casei*, *L. paracasei*, and *L. zeae*. She presented vegetations on the tricuspid valve and the right atrial lead and underwent catheter-based thrombectomy and a removal of the AICD. The removed material was positive for lactobacilli and, after six weeks of antibiotic therapy with multiple negative blood cultures, the patient did not show cardiac vegetation anymore. The patient reported habitually taking a lactobacillus probiotic [20].

*L. paracasei* and *L. plantarum* were involved in six cases of bacteremia in pediatric hematopoietic cell transplant recipients who received probiotic blends. The identity of the strains isolated from blood and from the probiotic preparations was confirmed using WGS [21].

Bacteremia induced by the probiotic bacterium *L. casei* was reported in a 63-year-old patient after the attempted removal of implantable cardioverter–defibrillator (ICD) electrodes, complicated by tricuspid valve damage and replacement. After the intervention, the patient required intensive care treatment with mechanical ventilation, continuous renal replacement therapy, broad-spectrum empirical antibiotic therapy, parenteral nutrition, and blood transfusions because of multiple organ failure. After 14 days, the patient developed septic shock. *L. casei* was isolated from the dialysis catheter. Based on antibiotic susceptibility of the isolate, piperacillin–tazobactam and linezolid therapy was initiated, and the patient improved. The possible source of infection was the Actimel Danone^®^ product, including *L. casei*, that the patient regularly consumed [22].

Endocarditis involving the native valves in a 71-year-old woman immunocompromised for rheumatoid arthritis treatment with prednisone was caused by *L. casei*. The patient declared that she took 2 g of an over-the-counter probiotic containing *L. casei* daily for several months. After treatment with ampicillin and daptomycin, she underwent a replacement for both the aortic and mitral valves [23].

A case of *L. paracasei* bacteremia complicated by native valve endocarditis and embolic cerebrovascular infarct regarded a 56-year-old immunocompetent man. He presented dyspnea, aortic sclerosis, and diastolic dysfunction. *L. paracasei* grew in the blood cultures that were later negative, so he was not treated with antibiotics. However, after two months, he experienced dysarthria and was found to have an embolic stroke, for which he was discharged with antiplatelet therapy. After three months, he presented acute dysarthria due to stroke recrudescence and *L. paracasei* grew in the blood cultures again. The patient mentioned that he had taken probiotics daily one year before for diarrhea and he consumed yogurt daily. He presented multiple dental caries, a known predisposing factor for lactobacilli infections. A transesophageal echocardiogram revealed an aortic valve vegetation and a calcified nodule, indicating old vegetation that led to researchers to suspect that the strokes were caused by emboli deriving from the valvular vegetation. Finally, he was treated with ampicillin and underwent valve replacement [3]. 

### 2.13. Lactococcus spp.

The genus *Lactococcus* comprises technologically relevant homofermenting LAB bacteria, with *L. lactis* and *L. cremoris* as the most widely applied in dairy fermentations. Among the lactococci, *L. garviae* has been recognized since 1950 as a fish pathogen affecting aquaculture establishments. It becomes part of the human intestinal microbiota after raw fish ingestion and can behave as an opportunistic pathogen in immunosuppressed individuals as well as infect cardiac protheses in immunocompetent patients. 

Recently, it was the cause of recurrent tonsillitis in two young males of 15 and 8 years of age. It was suggested that the infectious agent established in the tonsillar crypts after ingestion by forming a biofilm. *L. garvieae* was isolated from the tonsillar exudate in both cases and showed multi-resistance to penicillin, cephalosporins, macrolides, and quinolones. Based on these results, a combination of amoxicillin–clavulanic acid coupled with a sublingual bacterial autovaccine containing an inactivated suspension of the pathogen was successfully used as treatment [24]. 

This bacterium was also a cause of infectious endocarditis favored by graft placement in a 65-year-old man, who was hospitalized for headache and dysarthria caused by an intracranial aneurysm. *L. garviae* was isolated from the blood cultures and the patient was treated with intravenous antibiotics, ceftriaxone, and gentamycin [25]. 

In a 77-year-old man admitted to the hospital with back pain that had lasted for 5 days, *L. garvieae* was found to be the cause of spondylitis. The patient had diabetes mellitus, hypertension, and a history of pulmonary tuberculosis and abdominal aortic aneurysm repair. *L. garvieae* grew in the blood cultures and was identified using MALDI-TOF MS. The condition worsened because of an insufficient duration of the first antibiotic treatment and was resolved after six months of treatment with levofloxacin. The most probable access of *L. garviae* into the bloodstream can be the gastrointestinal tract in case of physiologic defects and a loss of integrity. However, this patient, who was exposed to raw fish, did not suffer from gastrointestinal diseases [26].

The species *L. lactis* caused cholangitis in a 70-year-old man with distal cholangiocarcinoma and bile duct obstruction. An anaerobic blood culture turned positive for Gram-positive cocci identified as *L. lactis* using MALDI TOF MS. The bacteremia originated from cholangitis and the clinical course after initiating the antibiotic treatment was good. However, the patient died because of metastatic cancer. Only another case report of *L. lactis*-associated cholangitis and bacteremia was described previously in a female patient and was the consequence of the presence of a bile duct stone. The colonization of *L. lactis* in the digestive tract and the occurrence of biliary obstruction are considered to be the two predisposing factors for this rare condition. *L. lactis* colonization of the digestive tract is occasional and occurs after the consumption of foods containing this bacterial species [27]. 

### 2.14. Laribacter hongkongensis

*Laribacter hongkongensis* is a Gram-negative, S-shaped bacillus of the Chromobacteriaceae family, class β-proteobacteria, first isolated from the blood and thoracic empyema of a patient with alcoholic liver cirrhosis in Hong Kong. It is associated with community-acquired gastroenteritis and traveler’s diarrhea and was found in about 60% of the intestines of commonly consumed freshwater fish of the carp family. 

A recent case of bacteremia caused by *L. hongkongensis* occurred in a 31-year-old patient with end-stage alcoholic cirrhosis. Similar to the other cases of invasive and non-invasive *L. hongkongensis* infections, the gastrointestinal tract was likely the portal of entry, as in three other cases described previously. All the patients suffered from severe liver disease that favored *L. hongkongensis* translocation through the gastrointestinal mucosa due to portal venous congestion, vasculopathy that can cause edema of the intestinal mucosa, and local immunosuppression [28]. 

### 2.15. Leuconostoc spp.

Leuconostocs are heterofermentative LAB associated with fermented dairy products and vegetables, but rarely associated with serious human infections. Most cases of human illness caused by *Leuconostoc* spp., such as peritonitis and endocarditis, occurred in immunocompromised patients or patients with central venous catheters or endotracheal tubes; however, a few occurred also in immunocompetent patients. Cases have also been reported in association with short gut syndrome, gastrointestinal procedures, and preterm birth. 

A recent case regarded a 30-year-old man with a history of papillary thyroid cancer and hepatitis C infection, who presented stabbing chest pain with radiation to the jaw. He reported that he had partially curdled raw milk from a friend’s farm some weeks before hospitalization and had already completed four days of antibiotic therapy. The blood cultures showed the growth of Gram-positive cocci but did not originate the colonies, so *L. mesenteroides* subsp. *cremoris* was identified directly in the blood cultures using MALDI-TOF MS. Due to the intrinsic resistance of *Leuconostoc* spp. to vancomycin for the presence of D-lactate instead of D-alanine in peptidoglycan, a 7-day course of antibiotics with daptomycin was undertaken. The next blood cultures were negative. The predisposing condition for this case was considered to be poor dentition [29]. Indeed, *Leuconostoc* spp. have been isolated from one acute facial cellulitis and an acute apical periodontitis [91]. 

### 2.16. Pantoea spp.

*Pantonea agglomerans*, previously denominated *Erwina herbicola* and later *Enterobacter agglomerans*, is ubiquitous in the environment and can contaminate plants and foods. It causes local or systemic opportunistic infection that can be severe in newborns and in the elderly. Neonatal cases and outbreaks were often nosocomial, with the main risk factors including an immunocompromised state due to an immature immune system and comorbidities [30]. Infant formula can be a food source of infection [92].

In a cross-sectional study carried out in the Nigerian city of Zaria, the first community-acquired newborn infection cases in Sub-Saharan Africa were identified. *P. agglomerans* was isolated from eight among 94 neonates who presented LOS. None of them had comorbidities, invasive procedures, intravenous catheterization, maternal risk factors for infection, or a polymicrobial infection. All the neonates were treated with Ampiclox and gentamicin and one was changed to ciprofloxacin after 7 days. The mortality rate was 12.5%. The source of infection could not be infant formula, since all the babies were breastfed, so it was suspected that the hands of family members and the household environment served as the sources [30]. 

### 2.17. Pediococcus spp.

Pediococci are coccoid-shaped homofermenting LAB present in a variety of fermented foods and beverages that have both technological and probiotic relevance. However, some cases of infection caused by these bacteria were reported. A recent case of *Pediococcus* bacteremia occurred in a 16-year-old male who developed dasatinib-induced hemorrhagic colitis during maintenance therapy for leukemia. He was eating yogurt almost daily. Gram-positive cocci in pairs were isolated from the aerobic blood culture and in tetrads from the anaerobic blood cultures after 24 h of incubation at 37 °C. An analysis using MALDI-TOF MS identified the pathogen as *P. acidilactici*. Since the patient’s general condition was improving, antibiotic therapy was not started. The pathogenesis most probably began for the disruption of the gastrointestinal mucosa by hemorrhagic colitis, resulting in transient bacteremia [31]. 

### 2.18. Plesiomonas shigelloides

*Plesiomonas shigelloides*, previously *Aeromonas shigelloides* [32], is the only species in the genus *Plesiomonas*, which is allocated in the Enterobacteriaceae family. It is a cause of gastroenteritis with a higher frequency in summer in the tropical or subtropical regions. Transmission most often occurs from the consumption of contaminated seafood, commonly shellfish, uncooked food, or contaminated water [33]. In immunocompromised patients and patients with underlying conditions, *P. shigelloides* can cause bacteremia, cholangitis, cellulitis, and abscesses. In neonates, *P. shigelloides* can cause meningitis. The case fatality rate of severe infections caused by this bacterium is 40% (17/43) [34]. 

In a 75-year-old patient with chronic lymphocytic leukemia and diarrhea caused by *P. shigelloides* lasting for four weeks, a colonoscopy revealed multiple ulcers in the colon and final ileum. To the patient, oral ciprofloxacin was administered for 5 days with the complete resolution of diarrhea. *P. shigelloides* in this patient was identified by the gastrointestinal (GI) multiplex molecular panel (Newfoundland and Labrador Public Health Laboratory). The patient reported that he usually ate raw oysters for many years [32,33].

A case of newborn meningitis with a brain abscess and septicemia caused by *P. shigelloides* via the transplacental route was diagnosed in Prato, Italy, and was linked to the consumption of oysters by the mother one week before delivery. The prompt recognition of the infectious agent in this case soon allowed for an appropriate antibiotic treatment, excluding ampicillin and penicillin to which the bacterium was resistant, and the survival of the patient [32].

A 49-year-old man previously diagnosed with alcoholic cirrhosis was hospitalized for severe gastroenteritis, abdominal pain, and generalized weakness. The evening before developing the symptoms, he had eaten a “Dojo nabe” hotpot containing freshly caught loaches, some of which appeared undercooked. Twenty other people with no medical problems had eaten the hotpot with him and none of them developed symptoms. Infective enterocolitis was ascertained and Gram-negative bacilli, identified as *P shigelloides* using MALDI-TOF MS, grew in the blood cultures. *P. shigelloides* is known to occur in the intestine of loaches, that was the most probable source of the infection. The patient fully recovered from multi-organ dysfunction after treatment with Meropenem, later switched to cefotaxime and levofloxacin. People with cirrhosis are thought to be susceptible to *P. shigelloides* infection through chronic iron overload beyond an increased intestinal permeability [34].

### 2.19. Proteus mirabilis

*P. mirabilis* is a Gram-negative swarming bacterium that is frequently found in the environment and is part of the human and animal microbiota. It is considered a commensal, but many reports highlighted its potential as an opportunistic pathogen mainly involved in urinary tract infections (UTIs). In a study regarding its presence in meat, *P. mirabilis* was isolated from all chicken samples, and in 27.80% and 23.05% of beef and pork samples, respectively. Based on the presence of virulence genes, enterobacterial repetitive intergenic consensus (ERIC) fingerprinting, and a strong biofilm forming capacity, the strains from the UTIs and meat appeared to be closely related, reinforcing the hypothesis that meat can be considered an important source of *P. mirabilis* in the community [93].

Strains of *P. mirabilis* have been isolated in hospitals from patients suffering from sepsis, food poisoning, peritonitis, and meningitis. In recent years, a rise in food poisoning outbreaks with clinical symptoms like abdominal pain, diarrhea, nausea, and dizziness associated with *P. mirabilis* was reported worldwide. Epidemiological investigations indicated that meat products, bean products, fish, and cold dishes are commonly associated with food poisoning caused by *P. mirabilis* [94]. 

### 2.20. Sarcina ventriculi

*Sarcina ventriculi*, formerly *Clostridium ventriculi*, is a rarely occurring, non-motile, anaerobic, Gram-positive coccus with a carbohydrate fermenting metabolism. It survives and grows in acidic environments and is associated with delayed gastric emptying. It may be responsible for dyspepsia, abdominal pain, gastric ulcers, and rare emphysematous gastritis that can lead to gastric perforation. *S. ventriculi* is a commensal organism found in the soil and feces of humans, especially in those following a vegetarian diet. It was not ascertained whether it naturally colonizes the human intestinal tract or if its presence is due to contamination from food intake [95]. The organism appears to be innocuous in a stomach with intact mucosa and normal emptying, but proliferation and gastritis can occur in patients with mucosal injury from diabetic gastroparesis, surgery, or neoplastic obstruction [35].

One case regarded a 67-year-old woman with a long-standing gastroesophageal reflux that worsened over several months. She presented diffuse esophagitis with ulcerations and whitish patches interspersed between the ulcerations. Biopsies from the esophagus and duodenum showed the presence of Gram-positive cocci in the tetrads. Therefore, *S. ventriculi* infection was diagnosed according to the method currently accepted to obtain a diagnosis of this infection, that is microscopic observation. The patient was treated with metronidazole and pantoprazole and had an almost complete resolution [36].

In a 35-year-old patient with liver and kidney transplantation for primary sclerosing cholangitis and diabetic nephropathy due to type 1 diabetes, pharmacologically immunosuppressed, severe epigastric pain, nausea, vomiting with episodes of coffee ground-like emesis, and diarrhea occurred. Biopsies of the stomach showed areas of inflamed mucosa, areas with complete mucosal/submucosal necrosis, and abundant clusters of *S. ventriculi* in the exudates. Treatment with piperacillin–tazobactam and clindamycin led to an apparent improvement of gastritis. However, the patient died due to other complications because he could not comply with the immunosuppressive treatments before hospital admission due to financial problems [35].

### 2.21. Serratia marcescens

The genus *Serratia* comprises 23 species of Gram-negative bacteria that can be found in different environments. These organisms cause different human infections and are intrinsically resistant to many antibacterial agents. The type species of the genus, *Serratia marcescens*, is widespread in the environment and has the ability to cause a variety of infections in immunocompromised individuals, such as nosocomial wound, respiratory, and urinary tract infections; bacteremia; and meningitis with a possibly lethal outcome [96]. *S. marcescens* has repeatedly caused outbreaks in NICUs, where the sources of infection are colonized patients, the personnel hands, contaminated infant food, breast pumps and breast milk, medical devices, parenteral nutrition solutions, drugs, and care products [37,97]. 

Outbreaks of *S. marcescens* infections in NICUs can be caused by the contamination of breast milk. An outbreak was transmitted by contaminated breast pumps and stopped after changing the disinfection procedure. Three consecutive outbreaks in a NICU were caused by the cross-contamination of breast milk, and the colonization of neonates stopped after the reorganization of the procedures in the milk kitchen [37].

The pasteurization of donor milk at 62.5 °C for 30 min inactivates relevant pathogens, but the use of unpasteurized donor milk, though not recommended by most international guidelines, has been adopted in several German and Norwegian hospitals to preserve the immunological properties of raw donor milk and the presumptive better effects on child development. This practice can favor the spread of *S. marcescens* among patients by different means [37]. 

In an outbreak reported in 2019 in the University Hospital Magdeburg, Germany, an early phase in infant colonization was likely caused by unpasteurized donor milk. In this outbreak, 17 neonates were colonized and two developed bacteremia. One of these two, who was a preterm infant, developed meningitis and died. The culture-negative milk portions of the suspected donor, which probably contained viable *S. marcescens* below the detection limit, were given to the neonates and resulted in the colonization or infection of the other seven preterm infants. In a later phase of the outbreak, a fatal case of meningitis occurred because the infant temporarily shared the room with a colonized neonate. WGS highlighted the sequence identity for all the patient and breast milk isolates with the exception of one isolate with only a single nucleotide polymorphism (SNP), confirming the common origin of the isolates [37]. 

### 2.22. Shewanella spp.

There are currently 80 species classified in the genus *Shewanella*, class Gammaproteobacteria. Among these, *S. putrefaciens*, *S. algae*, and *S. xiamenesis* are recognized as human pathogens. They occur in fresh, marine, and sewage water mainly in moderate and warm climates and were also isolated from foods such as milk, cream, butter, eggs, poultry, raw fish or seafood, and beef. *S. putrefaciens* is a biofilm former and important spoilage agent for protein-rich refrigerated foods [98]. 

*S. putrefaciens* is a rare opportunistic pathogen associated with skin, soft tissue, and intra-abdominal infections, mainly biliary tract infections and peritonitis, for which cholelithiasis or liver cirrhosis are predisposing factors. Another major predisposing factor for infection with this pathogen is ESRD. *S. putrefaciens* can also cause bacteremia with possibly lethal courses. In addition, *S. putrefaciens* is able to invade human intestinal epithelial cells. Since *S. putrefaciens* infections are often polymicrobial, the pathogenic role of the bacterium has yet to be better clarified [98]. 

Neonatal and pediatric infections such as bacteremia by *S. putrefaciens*, sometimes lethal, were reported in infants with a low birth weight or preterm. Infective endocarditis developing in septic shock with multiple organ failure were reported mainly in predisposed individuals. In addition, diseases such as diabetes mellitus, peripheral vascular disease, malignant neoplasia, and immunosuppression, are underlying conditions that favor *Shewanella* infections, which are mostly community acquired and favored by low hygiene and exposure to and the consumption of contaminated seafood or fish [98,99].

Among the virulent determinants detected or overrepresented in clinical *Shewanella* spp. isolate genomes, several are related to the adherence, toxicity, swarming and swimming motility, and iron metabolism. Genes overrepresented in the genomes of clinical isolates include the peroxidase encoding gene *kat*G, previously reported to be an important factor for survival under oxidative stress [99].

A recent case report regarded an 84-year-old man with pancreatic cancer and liver metastases who presented a fever. He declared to have ingested raw fish several days before. Ceftriaxone was administered once and cefmetazole assumed for two weeks by the patient successfully treated the infection. A blood culture showed the growth of Gram-negative bacilli that were identified as *S. algae* using MALDI-TOF-MS and 16S rRNA gene sequence analysis [38]. 

An analysis of the cases of *Shewanella* infections in patients admitted to a regional hospital in Hong Kong in the years 2012 to 2020 showed that none of the uremic patients with peritoneal dialysis suffered from peritonitis. Three cases of bacteremia caused by *Shewanella* were observed in the patients with chronic kidney disease (CKD) and none had a vascular access. Two of the involved patients suffered from cholangitis, and one had necrotizing fasciitis. Therefore, the association between *Shewanella* infections and CKD was found not to be related to the modality of dialysis employed. However, it might be explained by the dysregulated iron homeostasis, resulting in an overall positive iron balance in CKD with iron binding to the siderophores produced by *Shewanella* species. The majority of *Shewanella* infections had not documented seawater contact; however, the habitual consumption of raw or undercooked seafood in Hong Kong could explain the relatively large number of local cases [39].

### 2.23. Streptococcus spp.

The genus *Streptococcus* comprises different groups of cocci-shaped Gram-positive bacteria able to cause opportunistic infections, with some groups strongly associated with foodborne illnesses [100]. 

*S. agalactiae* commonly colonizes human intestine and urogenital tracts and can cause invasive infections in neonates, pregnant women, immunocompromised patients, and persons with underlying diseases, such as type II diabetes or cancer. Most recently, it has been recognized as a foodborne pathogen responsible for infections, such as meningoencephalitis, septicemia, and septic arthritis, consequent to the consumption of traditional dishes prepared with raw fish in Southeast Asian countries [46,101]. An outbreak occurring in Singapore in 2015 involved the clone ST283, which caused invasive and systemic infections also in individuals with no underlying conditions [102]. This clone has been isolated in eleven countries on four continents and can be considered an emerging pathogen of wide diffusion. The time-calibrated phylogeny of 328 genomes from ST283 isolates collected between 1998 and 2021 indicated an isolate from 1982 as the most recent common ancestor [101]. 

A recent case report regarded two sisters of 58 and 55 years of age who contracted *S. agalactiae* ST283 after the consumption of traditional raw fish dishes in Lao PDR. The older sister manifested musculoskeletal pain, nausea, vomiting, and watery diarrhea, while the other sister had a fever and joint infection. The organism was isolated from the blood cultures already after 24 h from symptom onset. A more severe progress of the illness was probably prevented by prompt antibiotic treatment with ceftriaxone and gentamicin [40]. Two other recent cases associated with raw fish consumption were recorded in Malaysia and were the first reported in this country. One occurred in a 36-year-old Chinese man presenting septic arthritis with no comorbidities and no association with raw or undercooked food. The other was observed in a 74-year-old Chinese woman who had diarrhea and vomiting after a visit to a durian orchard two weeks before and who arrived at the hospital with symptoms of meningoencephalitis. In this case, there was also no association with raw fish consumption. WGS showed that the *two S. agalactiae* ST283 strains isolated from the patients differed by three single nucleotide polymorphisms (SNPs) from each other, and by only one and two SNPs from the isolates from the human sepsis cases in Singapore [103]. It was speculated that the increasing number of infections caused by *S. agalactiae* ST283 in fish might derive from water contamination from human sources and could be prevented by improving wastewater management [101].

*S. dysgalactiae* is a β-haemolytic streptococcus able to cause mastitis in milk-producing animals and a recognized zoonotic agent that can cause infection in humans through the ingestion of contaminated food. The related illnesses are mainly skin and soft tissue infections, including pyoderma, cellulitis, wound infections, abscesses, erysipelas, tonsillitis, infectious endocarditis, necrotizing fasciitis, and hematogenous complications of bacteremia. This bacterial species is susceptible to β-lactams but, to avoid delayed, poor responses or failure of antibiotic therapy, the addition of an aminoglycoside to penicillin/cephalosporins should be considered for severe infections [41,104]. Bloodstream infections with *S. dysgalactiae*, in which the subspecies *equisimilis* is most often involved, are frequent in the elderly and may lead to sepsis, septic shock, and complications such as symmetric peripheral gangrene, which can require limb amputation [101,105]. 

A retrospective study regarding the years 2006 to 2020 carried out in Finland, where infections caused by β-hemolytic streptococci must be mandatorily notified, showed that the incidence of invasive infections attributable to these bacteria are rising, together with those due to *S. agalactiae*, for patients older than 55 years. It also showed a higher percentage of relapses. This trend was also observed globally and was explained by an improvement in the identification tests, an increase in the older population with comorbidities, and the diffusion of epidemic clones [105,106]. One recent case of suspected food origin regarded a 77-year-old man who had a history of unpasteurized milk consumption and presented a periprosthetic joint infection in one knee that became swollen and hyperemic. *S. dysgalactiae* was isolated from the knee aspirate. The case was resolved surgically and through antibiotic treatment [41].

Another zoonotic *Streptococcus* species involved in foodborne infections is *S. equi* subsp. *zooepidemicus* (SEZ), a highly contagious opportunistic pathogen found in horses and other farm animals. Human infections are considered rare and may occur in persons who are exposed to animals or consume raw horse meat and unpasteurized milk [42]. Clinical manifestations include meningitis, sepsis, peritonitis, necrotizing myositis, purulent arthritis, purulent pericarditis, and endocarditis [107]. Of three cases registered in Jeju Island, South Korea, between 2009 and 2019, one regarded a 59-year-old man with hypertension and end-stage renal disease on dialysis. The infection was probably caused by the consumption of raw horse meat and liver and manifested as a joint infection in both knees evolving into septic arthritis. The patient recovered after the drainage of the joint fluid, intravenous levofloxacin treatment, and rehabilitation [42]. 

Another case regarded a 49-year-old woman with liver cirrhosis who manifested abdominal pain and edema of the extremities four days after eating raw horse meat. She developed SEZ bacteremia and healed after treatment with teicoplanin and ceftriaxone. A review of the literature showed that among the cases of SEZ infections occurring in other countries, 19 were linked to the consumption of raw horse meat or unpasteurized milk, and foodborne infections showed higher mortality than those caused by contact with horses [42].

A retrospective study for the years 2005 to 2020 carried out in Thailand identified 18 cases of SEZ infection, with septicemia in 61% cases and 72% linked to raw pork consumption. The isolates belonged to ST194 and those from different patients had identical pulsotypes. Based on the SNP phylogenetic analysis, the clinical strains were closely related to the swine ST194 strains, which were reported to cause high-mortality infections with sudden death in pigs from China, Canada, and the U.S.. Other STs of clinical importance were STs 5, 10, 65, 72, 209, 306, and 364 [108].

Two infection reports attributable to the consumption of unpasteurized milk and derived products have been published since 2019. One regarded a 73-year-old woman with an osteodural defect, chronic otitis, and other underlying diseases who developed meningitis. The other case regarded bacteremia in a newborn and her 31-year-old mother, who consumed contaminated artisanal cheese five days before the baby’s birth [43,44].

A recent large outbreak, with 37 cases, occurred in Central Italy in the period of November 2021 to May 2022 and was traced back to the consumption of cheese made from unpasteurized milk from a single manufacturer [45]. The patients manifested different infective states, including septicemia, pharyngitis, arthritis, uveitis, endocarditis, and meningitis. Five patients who developed meningitis died. Based on an SNP analysis of the genomes, 21 clinical isolates were closely related, indicating a single source of infection. The epidemiological investigation of the outbreak led researchers to ascertain that 31 patients had consumed soft cheeses from local producers. The inspection of eight of the producers led to the identification of one cheese manufacturing plant contaminated by *S. equi* subsp. *zooepidemicus*, which was found to be present in both raw milk and raw milk cheese. The isolates from the producer clustered with the clinical ones and with an isolate from cow mastitis were obtained in November 2021 and originated from the same farm. The reported outbreak indicated that SEZ is an important emerging pathogen with a high virulence and that the use of WGS coupled with epidemiological investigation can prevent further infection cases [45].

Bacteria belonging to the *Streptococcus bovis/Streptococcus equinus* complex (SBSEC) colonize the intestinal tract of both humans and animals and can behave as opportunistic pathogens if they cross the intestinal barrier that can be damaged by diseases or medical procedures. These bacteria can cause bacteremia and localized infections, more often endocarditis but also peritonitis [105], meningitis [109], cholecystitis [46], and pancreatitis [110]. 

Moreover, a strong association between *S. bovis*, mainly biotypes I and II/2, reclassified as *S. gallolyticus* [111], and colorectal cancer (CRC) has been known since 1977. Therefore, the Infectious Diseases Society of America (IDSA) has recommended that patients with SBSEC bacteremia should be evaluated for CRC. Some theories suggest that these bacteria favor the development of malignant lesions from premalignant ones by inducing inflammatory responses and mutations in tumor suppressor genes [100]. 

Though it was hypothesized that the dietary route favors intestinal colonization by these bacteria, no case reports or outbreaks have been linked to food except for one, alluding to a possible food origin of the infectious agent and involving *S. gallolyticus* subsp. *pasteurianus*. This case regarded a 63-year-old man with a history of severe gastroenteritis many years before, who presented burning epigastric pain and vomiting due to acute necrotizing cholecystitis that started two hours after the ingestion of a pork cutlet. The gallbladder contained more than 30 dark brown bilirubin calcium stones between 2–3 mm in diameter covered with bacterial colonies. The colon did not present malignant lesions. The case was successfully treated surgically and through antibiotic administration [46]. 

*S. iniae* is another pathogen that causes infections in aquatic animals and is of great concern in aquaculture. However, it is also capable of causing bacteremia cellulitis, arthritis, meningitis, and endocarditis in humans [112]. No case reports related to food consumption seem to have occurred recently.

*Streptococcus suis* causes infectious diseases that can be transmitted to humans by direct contact with sick pigs or the ingestion of contaminated meat, resulting mainly in meningitis but also septicemia, pneumonia, toxic shock, arthritis, endocarditis, and endophthalmitis. The most frequent sequela is hearing loss, occurring in more than 50% of cases [47]. Having a pig-related occupation and male sex are risk factors for infection by *S. suis* [48]. 

In recent years, the number of reported human *S. suis* cases has increased, mostly in Southeast Asian countries. While *S. suis* zoonosis is more of an occupational disease affecting workers in close contact with infected pigs or contaminated pork in industrialized countries, in Southeast Asia it is more linked to foodborne infections. Indeed, high pig densities and the consumption of raw or undercooked pork has determined more than 50% of the total human *S. suis* cases in Asia. This important zoonotic pathogen has been classified into 29 serotypes. Serotype 2 is most frequently recovered from human infections, although human cases due to serotypes 4, 5, 7, 9, 14, 16, 21, 24, and 31 have also been reported, with a higher diversity in Southeast Asia. A study from Vietnam showed significant problems with mobility, self-care, the performance of usual activities, and the emotional impact caused by hearing impairment and dizziness sequelae from *S. suis* infections, also with a sanitary burden. The infection spread in Southeast Asian countries is favored by the lack of proper identification for infected animals, poor meat inspection, and limited access to hygiene measures when handling raw pork during slaughter or in kitchens. Consequently, *S. suis* can contaminate working surfaces and the hands of the operators in the whole pig supply chain, from slaughterhouses to retail markets [113]. 

Four large outbreaks of *S. suis* infections in humans have been recorded in Thailand, mainly in the North [114]. The incidence of *S. suis* disease in Thailand peaked during the rainy season, and an association was found between the occurrence of human *S. suis* meningitis and porcine reproductive and respiratory syndrome virus (PRRSv) outbreaks on pig farms. More than 70% of cases with *S. suis* infections were associated with the consumption of traditional raw pork and raw pig blood dishes but also of a cooked pork traditional dish in Vietnam, thus indicating cross-contamination events. Though prevalence data were not available, human *S. suis* meningitis cases were reported in Indonesia, Lao PDR, and the Philippines, all countries with a tradition of raw pork consumption [115]. To evaluate the genotypic relationship between the *S. suis* isolates recovered from either human or pig origins, *S. suis* isolated from pig tonsils at a slaughterhouse in Phayao province between April 2010 and March 2011 were studied and compared to the human isolates recovered in the same region. Thirteen out of 17 serotype 2-ST1 isolates, five out of seven isolates of serotype 2-ST25, one out of four isolates of serotype 2-ST28, and all serotype 2-ST103 and ST104 isolates revealed pulsotypes identical to those of the human isolates [115].

*S. suis* infection is endemic in China, owing to frequent pork consumption and small-scale swine farming. Although human *S. suis* is normally presented as a sporadic disease, there were two outbreaks in China with toxic shock syndrome as the most severe presentation. The fatality rate of human *S. suis* infection in China was up to 18% [47]. 

A recent case in Shandong province involved a 75-year-old previously healthy man who presented a 1-day history of a fever, vomiting, coughing, chills, and unconsciousness and received a diagnosis of sepsis and intracranial infection. Gram-positive cocci able to form small colonies were identified as *S. suis* grew in the blood cultures. Based on antibiotic susceptibility testing, the patient was treated with levofloxacin and recovered, though with hearing loss. It was ascertained that the patient had eaten pork from a sick pig before disease manifestation [47]. 

A 48-year-old man, who often consumed raw fermented pork presented endophthalmitis in the right eye that evolved into a perforated cornea and vitreous hemorrhage. Two days later, he developed a low-grade fever, neck, joint and waist pain, and blurred vision in his right eye along with a decrease in hearing in the right ear. His physical weakness from sleep deprivation and underlying conditions were probably the predisposing factors for the infection. *S. suis* was detected in the blood cultures and vitreous from the right eye. The audiogram was done at 5 days post-admission, suggesting that irreversible bilateral sensorineural hearing loss (SNHL) resulted from the disseminated *S. suis* infection [48]. 

A case of meningitis in a Brazilian elderly male occurred after the consumption of pork and was successfully treated with ceftriaxone. Another case reported in Brazil regarded a 49-year-old woman who was probably infected by contact with pork contaminated with *S. suis* while cooking and who suffered meningitis and bacteremia [49,50]

### 2.24. Weissella Confusa

Among the 22 recognized species of the heterofermentative LAB genus *Weissella*, *W. confusa* is the most frequently associated with human infections, more often with bacteremia. It has also been isolated in co-infections. The majority of serious infections caused by this bacterial species have been reported in immunocompromised patients with comorbidities. Generally, translocation from the gut is the most common mode of infection, especially in immunocompromised but also in immunocompetent individuals and individuals with prolonged hospital stays where multi-drug-resistant clones are selected [116]. 

On the other hand, *W. confusa*, which is associated with different foods, mainly fermented plants, was proposed to be safe for food production and as a probiotic [117]. 

A case of *W. confusa* infection was diagnosed in a 11-year-old male child admitted to the hospital with a high fever, difficulty breathing, tachycardia, and tachypnea. He was diagnosed with acute pancreatitis with severe inflammatory response syndrome (SIRS) and evolving acute respiratory distress syndrome (ARDS). He was treated via percutaneous draining. From a blood culture, Gram-positive cocci identified as *W. confusa* were isolated. A co-infection with *C. parapsylopsis* was revealed and piperacillin–tazobactam, ceftazidime, and voriconazole were administered, leading to patient recovery [113]. 

A 65-year-old man with alcohol-associated cirrhosis, and currently undergoing liver transplant evaluation, arrived at the hospital with confusion, weakness, and weight loss. He had a negative chest X-ray and urinalysis, but the blood cultures grew Gram-positive rods, identified as *W. confusa*, in less than 24 hours. He reported eating sauerkraut on a weekly basis for several years and had no new food exposures. The repeat blood cultures were negative but aortic valve infectious endocarditis was diagnosed. The patient was treated with penicillin V followed by oral amoxicillin–clavulanate and he recovered after some complications. Another case of *W. confusa* infection attributed to the consumption of sauerkraut was previously reported [51].

In a 63-year-old man of Ghanaian origin, who suffered chest tightness and palpitations for four weeks, the blood cultures were positive for Gram-positive coccobacilli identified as *W. confusa* using MALDI-TOF MS. Therefore, intravenous amoxicillin and ceftriaxone were administered. A transthoracic echocardiogram revealed a thickened bicuspid aortic valve with a suspicion of mobile vegetation. He needed an aortic valve replacement, and the debrided aortic material was negative for bacteria, so antimicrobial therapy was not restarted. The patient was discharged after a full recovery on day 62 of his admission. *W. confusa* is used in the fermenting process of commonly consumed Ghanaian food products, such a “nunu”, a yoghurt-like product prepared from raw cow’s milk, and it was considered possible that the patient’s Ghanaian diet contributed to gut colonization with *W. confusa* [52].

A 78-year-old male patient, bedridden due to an old stroke and many comorbidities and with a history of infectious states, presented a high fever and an altered mental status for 2 days. He was lethargic, responsive only to painful stimuli, and had generalized stiffness. The CSF was turbid but with no microorganisms. The blood cultures were positive for Gram-positive cocci in pairs and chains that were identified as *W. confusa* using MALDI-TOF MS. These were also found later in the CSF. Vancomycin, to which *W. confusa* is intrinsically resistant, was then stopped and the patient was treated with meropenem and ampicillin, returning him to his previous health status. Age, diabetes, and a prior history of cholangitis, a manipulation of the biliary tract, and possible intestinal micro-perforations might have contributed to the translocation of *W. confusa* to the bloodstream causing bacteremia with secondary meningitis. The diet of the patient consisting of mashed fruits and vegetables for the past few weeks might also have played a role, given the frequent occurrence of *W. confusa* in vegetables [53].

## 3. Conclusions

This review was based on outbreak or case reports that only account for a small part of the illnesses caused by opportunistic pathogens and transmitted via the dietary route. However, it gathers the risk factors and indications for the prevention of pathogens belonging to different taxonomic groups. Only a few of the opportunistic pathogens considered in the survey are an object of surveillance by public health authorities, and those few are so only in certain countries. This most probably contributed to the lack of prevalence data and the missed adoption of specific prevention measures, including the correct information of the consumers on the risks linked to the food products involved when associated with particular medical conditions. 

For some of the opportunistic pathogens considered in this survey, e.g., *Acinetobacter* spp., *Chryseobacterium* spp., CNS, *Klebsiella pneumoniae*, and *Enterococcus* spp., the focus of the investigations was centered mainly on nosocomial infections and outbreaks caused by multi-drug-resistant clones selected in clinical settings whose origin was most often not identified. However, the occurrence of those bacterial genera and species in food or water is very frequent. Therefore, the study of the possible connections between food and drinking water consumption and environmental or host colonization should be intensified to allow for the interruption of the chain of the contamination events leading to illnesses. Monitoring the nosocomial environment for the presence of all the opportunistic pathogens, whose association with hospital-acquired infections is well established, should become a routine practice aimed at reducing suffering for patients and saving lives.

Some of the opportunistic bacterial pathogens considered in this review, namely, *S. epidermidis* and *S. marcescens* in particular, but also *K. pneumoniae*, *K. aerogenes*, and *P. agglomerans* were associated with infections and outbreaks in NICUs. While the dietary origin of *S. epidermidis* in NICU infections seems to be excluded, the same cannot be stated for the other pathogens for which breast milk or formula can be involved. The use of appropriate techniques, such as sensitive molecular methods, to analyze these food sources before administration could allow for an efficient prevention of neonatal infections.

Lactobacilli was the bacterial group with the highest number of infection cases reported in the current year. This can be explained by the most frequent exposure of persons to a high number of these bacteria through the intake of probiotic preparations and fermented milks. Therefore, it is opportune to select the strains devoid of possible virulence traits more carefully for use as probiotics and for the production of fermented food products. The same can be stated for the other LAB involved in food fermentation and considered in this review, namely the *Lactococcus*, *Leuconostoc*, *Pediococcus*, and *Weissella* species.

In many of the reports surveyed here, the pathogenic agent was not characterized using molecular typing methods. This constituted a relevant obstacle to the identification of the food sources involved in the transmission and the source of contamination. Therefore, the introduction of practices that facilitate clinical isolate typing by specialized laboratories might aid in the reduction in foodborne illness cases by focusing the surveillance on specific clones. This could also lead to the development of molecular methods that could be applied in food safety control.

The AR profiles of the isolates causing infections were explicitly mentioned only in 15 out of the 49 reports listed, and in most cases, antibiotic therapy was initiated empirically before the identification and antimicrobial resistance testing of the isolate. This was commonly done since most clinical laboratories rely on the culture-based identification of bacteria after their isolation. This may require at least one day to obtain the results, while the patient requires immediate care. The use of molecular panels to identify the bacteria present directly in a clinical specimen and the corresponding AR genetic profile would be highly beneficial to initiate the most appropriate therapy and should be made available for clinicians by connecting equipped diagnostic institutions.

Finally, it was highlighted that traditions of raw food consumption are implied in the occurrence of infections in some countries. This points out the necessity to improve hygiene practices and increase microbiological testing of foods before commercialization for opportunistic pathogens beyond major pathogens. 

## Figures and Tables

**Table 1:** Food products involved in the transmission of opportunistic bacterial infections, bacterial taxa implied, and related illnesses.

Dietary Source	Infectious Agent and Related Illness
Artisanal cheese	*Streptococcus equi* subsp. *zooepidemicus* perinatal bacteremia [44]; different infections in 37 patients [45]
Breast milk	*Klebsiella pneumoniae* bacteremia [14]; *Serratia marcescens* bacteremia [37]; *Pantoea agglomerans* neonatal late onset sepsis (LOS) [30]
Chicken	*Arcobacter* spp. pericarditis [6]
Composite dishes	*Bacillus pumilus* bacteremia [10]; *S. agalactiae* septic arthritis and bacteremia [40]
Drinking water	*Enterococcus faecalis* gastroenteritis [13]; *K. aerogenes* urinary tract infection (UTI) [15]
Fermented pork	*S. suis* bacteremia, endophthalmitis [48]
Yogurt	*Lacticaseibacillus paracasei* endocarditis, embolic cerebrovascular infarct [3], cholecystitis [16], septic shock [22]; *Lactobacillus jensenii* endocarditis [19]; *Pediococcus acidilactici* bacteremia [31]; *Weissella confusa* endocarditis [52]
Natto (fermented soybeans)	*B. subtilis* (natto) bacteremia [9]
Pork cutlet	*Streptococcus gallolyticus* subsp. *pasteurianus* acute necrotizing cholecystitis [46]
Probiotic preparation	*Bacillus licheniformis* bacteremia [8]; *L. rhamnosus* septic shock, endocarditis with brain emboli [17]; *L. paracasei* endocarditis, embolic cerebrovascular infarct [3], lumbar osteomyelitis [18] and bacteremia [21] (probiotic preparation or yogurt) [3]; *Lacticaseibacillus* spp. endocarditis [20]; *L. casei* endocarditis [23]
Raw fish and seafood	*Aeromonas hydrophila* phlegmonous gastritis, mild renal failure, sepsis [5]; *Lactococcus lactis* spondylitis [26]; *Plesiomonas shigelloides* ulcers in colon and final ileum [32], meningitis, brain abscess, septicemia in a newborn [33] and septic shock [34]; *Shewanella putrefaciens* bacteremia [38]
Raw horse meat	*S. equi* subsp. *zooepidemicus* septic arthritis, abdominal infection, edema of the extremities, bacteremia [42]
Raw pork	*S. suis* sepsis and intracranial infection [47], meningitis [49], meningitis, septicemia [50]
Sauerkraut	*W. confusa* septicemia, endocarditis [51]
Stinky tofu	*A. sobria* peritonitis [4]
Smashed vegetables	*W. confusa* bacteremia, meningitis [53]
Unpasteurized milk	*Leuconostoc mesenteroides* bacteremia [29]; *S. dysgalactiae* knee joint infection [41]; *S. equi* subsp. *zooepidemicus* meningitis [43]
Unknown food sources	*A. butzleri* prolonged watery diarrhea [7]; *Comamonas testosteroni* bacteremia [11]; *E. casseliflavus* bacteremia, liver cyst infection [12]; *L. garviae* recurrent tonsillitis [24] and endocarditis [25]; *L. lactis* bacteremia, cholangitis [27]; *Laribacter hongkongensis* bacteremia [28]; *Sarcina ventriculi* esophagitis with ulcerations [35] and mucosal/submucosal necrosis [36]; *S. putrefaciens* bacteremia [39]

## Data Availability

Not applicable.

## Supplementary Materials

The following supporting information can be downloaded at: https://www.mdpi.com/article/10.3390/microorganisms12010069/s1, Table S1: Opportunistic bacterial pathogens involved in foodborne severe infections reported since 2019, confirmed or most probable dietary sources, underlying conditions or predisposing factors, identification methods and antibiotic resistance (AR) profile of isolates.
