# Supplementary material for: Recent Records on Bacterial Opportunistic Infections via the Dietary Route
Source: Microorganisms. 2023 Dec 29;12(1):69. doi: 10.3390/microorganisms12010069 (PMC10819555; doi:10.3390/microorganisms12010069)
Supplement: Supplementary file 1 [file microorganisms-12-00069-s001.zip › microorganisms-2775094-supplementary.pdf]

**Table S1.** Opportunistic bacterial pathogens involved in foodborne severe infections reported since 2019, confirmed or most probable dietary sources, underlying conditions or predisposing factors, identification methods and antibiotic resistance (AR) profile of isolates.

| Infectious agent | Illness | Dietary source | Predisposing/ underlying condition | Identification technique | AR |
|---|---|---|---|---|---|
| *Aeromonas sobria* | peritonitis [4] | stinky tofu | peritoneal dialysis (PD) | not specified | ampicillin, cefotaxime, piperacillin /tazobactam |
| *A. hydrophila* | phlegmonous gastritis, mild renal failure, sepsis [5] | raw fish | gastric ulcer | not specified | levofloxacin[a] |
| *Arcobacter* spp. | pericarditis [6] | chicken | Human Immunodeficiency Virus (HIV) and Covid-19 infections, end-stage renal disease (ESRD) | Vitek 2 (bioMerieux) | none reported |
| *A. butzleri* | prolonged watery diarrhea [7] | unknown | HIV infection | not specified | none reported |
| *Bacillus licheniformis* | bacteremia [8] | probiotic | Intestinal bleeding | MALDI-TOF MS, whole genome sequencing (WGS) | levofloxacin[a], vancomycin[a] |
| *B. subtilis* (natto) | bacteremia [9] | natto | peritonitis | MALDI-TOF MS *bio*F and *bio*W gene regions sequencing | piperacillin/tazobactam[a], teicoplanin[a] |
| *B. pumilus* | bacteremia [10] | rice and meat dish | none | API 20E and API 50CHB (bioMerieux), 16S rRNA gene sequencing | cefepime, cefotaxime |
| *Comamonas testosteroni* | bacteremia [11] | unknown | none | routine physiological tests and VITEK-2 | piperacilline-tazobactam |
| *Enterococcus casseliflavus* | bacteremia, liver cyst infection [12] | unknown | hemodialysis | not specified[b] | vancomycin (moderate resistance), dalfopristin/quinupristin, |
| *E. faecalis* | gastroenteritis in 690 individuals [13] | drinking water | none | not specified[b] | none reported |

| | | | | | |
|---|---|---|---|---|---|
| *Klebsiella pneumoniae* | bacteremia [14] | breast milk | preterm birth | Vitek 2 | extended-spectrum cephalosporins, aztreonam, clavulanic acid synergy, nitrofurantoin, trimethoprim-sulfamethoxazole, tobramycin, gentamicin |
| *K. aerogenes* | urinary tract infection (UTI) [15] | water | type 2 diabetes, hypertension | Phenotypic tests and API 20E test (BioMérieux), WGS | aminoglycosides, penicillin, cephalosporins, amphenicol, fluoroquinolones, folate drugs, tetracyclines, phosphonic acid, glycycline[c] |
| *Lacticaseibacillus paracasei* | cholecystitis [16] | yogurt | type 2 diabetes, hypertension, cardiac problems, cholelithiasis | Vitek | metronidazole[a], ceftriaxone[a] |
| *L. rhamnosus* | septic shock, endocarditis with brain emboli [17] | probiotic | ulcerative colitis, type 2 diabetes, heart surgery, immunosuppression | MALDI-TOF MS | vancomycin[a], cefepime[a], azithromycin[a], meropenem |
| *L. paracasei* | lumbar osteomyelitis [18] | probiotic | Type 2 diabetes, ischemic heart disease | MALDI-TOF- MS | ampicillin |
| *Lactobacillus jensenii* | endocarditis [19] | yogurt | none | MALDI-TOF- MS | none determined |
| *Lacticaseibacillus* spp. | endocarditis [20] | probiotic | Type 2 diabetes, cardiomyopathy | not specified | ciprofloxacin[a], metronidazole[a], meropenem |
| *L. paracasei* *Lactiplantibacillus plantarum* | bacteremia [21] | probiotic | hematopoietic cell transplantation | WGS | none reported |
| *L. paracasei* | septic shock [22] | yogurt | Cardiac surgery, multi-organ failure | MALDI-TOF MS | meropenem, vancomycin[a], |
| *L. casei* | endocarditis [23] | probiotic | Prednisone immunosuppression | not specified | meropenem, vancomycin |

| | | | | | |
|---|---|---|---|---|---|
| *L. paracasei* | endocarditis, embolic cerebrovascular infarct [3] | probiotic or yogurt | Dental caries | | |
| *Lactococcus garviae* | recurrent tonsillitis [24] | unknown | none | not specified | penicillin, cephalosporins, macrolides, quinolones |
| | endocarditis [25] | unknown | Aortic graft placement | Polymerase chain reaction (PCR) | none reported |
| *L. lactis* | spondylitis [26] | raw fish | diabetes mellitus, hypertension | MALDI-TOF MS | penicillin, clindamycin |
| | bacteremia, cholangitis [27] | unknown | bile duct obstruction | MALDI-TOF MS | intermediate resistance to penicillin |
| *Laribacter hongkongensis* | bacteremia [28] | unspecified | end-stage alcoholic cirrhosis | MALDI-TOF MS, 16S rRNA gene sequencing, multilocus sequence typing (MLST) | none reported |
| *Leuconostoc mesenteroides* | bacteremia [29] | raw milk | thyroid cancer, hepatitis C, poor dentition | MALDI-TOF MS | none reported |
| *Pantoea agglomerans* | neonatal late onset sepsis (LOS) [30] | suspected milk contamination | none | Microbact 24E (Oxoid) | ceftazidime, ceftriaxone, amoxicillin-clavulanate[d], ampiclox[a,e] |
| *Pediococcus acidilactici* | bacteremia [31] | yogurt | hemorrhagic colitis | MALDI-TOF MS | not tested |
| *Plesiomonas shigelloides* | meningitis, brain abscess and septicemia in a newborn [32] | oysters | transplancental transmission | not specified | not tested |
| | Ulcers in colon and final ileum [33] | oysters | chronic leukemia | MALDI-TOF MS | none reported |
| | septic shock [34] | loach | alcoholic cirrhosis | MALDI-TOF MS | ampicillin |
| *Sarcina ventriculi* | esophagitis with ulcerations [35] | unspecified | gastroesophageal reflux | optical and transmission electron microscope (TEM) observation | none reported |
| | mucosal/submucosal necrosis [36] | unspecified | liver-kidney transplantation | optical microscope observation | none reported |

| | | | | | |
|---|---|---|---|---|---|
| *Serratia marcescens* | bacteremia [37] | unpasteurized donor milk | preterm birth | MALDI-TOF MS, WGS | none reported |
| *Shewanella algae* | bacteremia [38] | raw fish | pancreatic cancer | MALDI-TOF MS, 16S rRNA gene sequencing | unclearly stated |
| *Shewanella putrefaciens* | bacteremia [39] | unspecified | chronical kidney disease (CKD) cholangitis (two patients) necrotizing fasciitis (one patient) | MALDI-TOF MS | ceftazidime[f], cefoperazone-sulbactam[f], ciprofloxacin[f], piperacillin[f], imipenem-cilastatin[f] |
| *S. agalactiae* | septic arthritis and bacteremia [40] | traditional dishes | none | Vitek 2 | none reported |
| *S. dysgalactiae* | knee joint infection by [41] | unpasteurized milk | none | MALDI-TOF MS | none reported |
| *Streptococcus equi* subsp. *zooepidemicus* | septic arthritis [42] | raw horse meat and liver | ESRD | MALDI-TOF MS, 16S rRNA gene sequencing | none reported |
| | abdominal infection, edema of the extremities, bacteremia [42] | raw horse meat | liver cirrhosis | MALDI-TOF MS, 16S rRNA gene sequencing | none reported |
| | meningitis [43] | unpasteurized milk | osteodural defect, chronic otitis | not specified[b] | none reported |
| | perinatal bacteremia [44] | artisanal cheese | none | Vitek, MALDI-TOF MS | none reported |
| | different infections in 37 patients [45] | raw milk cheese | varying underlying conditions | MALDI-TOF MS, WGS | none reported |
| *Streptococcus gallolyticus* subsp. *pasteurianus* | acute necrotizing cholecystitis [46] | pork cutlet | past severe gastroenteritis | API 20 STREP (bioMérieux), WGS | none reported |
| *S. suis* | sepsis and intracranial infection [47] | pork | none | not specified | none reported |

| | | | | | |
|---|---|---|---|---|---|
| | bacteremia, endophtalmitis [48] | fermented pork | stress from sleep deprivation | not specified | none reported |
| | meningitis [49] | pork | old age | MALDI-TOF MS | none reported |
| | meningitis, septicemia [50] | pork | none | MALDI-TOF MS | clindamycin, erythromycin |
| *Weissella confusa* | septicemia, endocarditis [51] | sauerkraut | alcohol associated cirrhosis | MALDI-TOF MS | vancomycin, teicoplanin |
| | endocarditis [52] | yogurt | none | not specified | vancomycin[a] |
| | bacteremia, meningitis [53] | smashed vegetables | many comorbidities, suspected intestinal microperforations | MALDI-TOF MS | vancomycin[a] |

[a]deduced from clinical outcome;
[b]performed by an external laboratory;
[c]confirmed at genetic level;
[d]in two among eight neonates;
[e]in one among eight neonates;
[f]only one isolate was resistant.
